# Genetic Screening of *Candida albicans* Inactivation Mutants Identifies New Genes Involved in Macrophage-Fungal Cell Interactions

**DOI:** 10.3389/fmicb.2022.833655

**Published:** 2022-04-05

**Authors:** Pablo Godoy, Peter John Darlington, Malcolm Whiteway

**Affiliations:** ^1^Centre of Structural and Functional Genomics, Biology Department, Concordia University – Loyola Campus, Montreal, QC, Canada; ^2^Department of Pathology and Microbiology, Faculty of Veterinary Medicine, University of Montreal, Saint-Hyacinthe, QC, Canada; ^3^Perform Centre, Department of Health, Kinesiology and Applied Physiology, Montreal, QC, Canada

**Keywords:** *Candida albicans*, macrophages, immune response, engulfment kinetics, fungal morphology, plasma membrane, cell wall

## Abstract

*Candida albicans*, an important fungal pathogen of humans, displays different morphologies, such as yeast, pseudo-hyphae and hyphae, which are recognized unequally by phagocytic cells of the innate immune response. Once *C. albicans* cells invade host tissues, immune cells such as macrophages are attracted to the site of infection and activated to recognize, engulf and kill the pathogen. We have investigated this fungal cell-macrophage interface by using high-throughput screening of the *C. albicans* GRACE library to identify genes that can influence this interaction and modify the kinetics of engulfment. Compared with the wild-type (WT) strain, we identified generally faster rates of engulfment for those fungal strains with constitutive pseudo-hyphal and hyphal phenotypes, whereas yeast-form-locked strains showed a reduced and delayed recognition and internalization by macrophages. We identified a number of GRACE strains that showed normal morphological development but exhibited different recognition and engulfment kinetics by cultured macrophages and characterized two mutants that modified interactions with the murine and human-derived macrophages. One mutant inactivated an uncharacterized *C. albicans* open reading frame that is the ortholog of *S. cerevisiae OPY1*, the other inactivated *CaKRE1*. The modified interaction was monitored during a 4 h co-culture. Early in the interaction, both *opy1* and *kre1* mutant strains showed reduced recognition and engulfment rates by macrophages when compared with WT cells. At fungal germ tube initiation, the engulfment kinetics increased for both mutants and WT cells, however the WT cells still showed a higher internalization by macrophages up to 2 h of interaction. Subsequently, between 2 and 4 h of the interaction, when most macrophages contain engulfed fungal cells, the engulfment kinetics increased for the *opy1* mutant and further decreased for the *kre1* mutant compared with Ca-WT. It appears that fungal morphology influences macrophage association with *C. albicans* cells and that both *OPY1* and *KRE1* play roles in the interaction of the fungal cells with phagocytes.

## Introduction

Fungal infections are a significant threat to human health. The diploid commensal fungus *Candida albicans* represents an important pathogen worldwide, and Candidiasis is able to cause as high as 40% mortality in immunocompromised patients (Horn et al., 2009). As a common member of the human microbiota, *C. albicans* will interact with other microbes as well as with host tissues (Höfs et al., 2016). *Candida* can live as a commensal on mucosal tissues such as the oral cavity, where it remains generally harmless and can persist long term (Naglik et al., 2011). A key aspect that influences *C. albicans* success as either a commensal or pathogen is its relationship with the host immune response (Netea et al., 2008). Candidiasis, the clinically defined systemic fungal disease, occurs when the normal host–fungal equilibrium is disrupted by overgrowth of typically invasive hyphal cells. Different conditions can trigger this behaviour, such as immune suppression by chemotherapy or HIV infection, or in organ transplant patients with suppressed immune responses (Fidel, 2002). When any of these scenarios take place, changes in gene expression in coordination with morphological remodelling can occur in the fungal cells and facilitate the transition from commensal to pathogen (Gow et al., 2002; Hube, 2004; Sudbery et al., 2004).

In response to an increased fungal burden, the immune response typically involves leukocytes, present in different tissues, as the first line of defense (Cheng et al., 2012; Qin et al., 2016). The interface of *C. albicans* and phagocytes has been well documented as one of the most studied host-pathogen interactions (Urban et al., 2009; Lewis et al., 2012; Erwig and Gow, 2016). Once the early immune response is triggered by the fungal cells, the first responders to the insult that arrive to control and clear the fungus correspond to leukocytes such as neutrophils and macrophages (Romani, 2011; Jimenez-Lopez and Lorenz, 2013). These phagocytic immune cells detect the presence of *C. albicans* by recognizing the pathogen’s surface structures, such as the cell wall, which contains several molecules that act as pathogen-associated molecular patterns (PAMPs). This initiates the induction of pattern recognition receptors (PRRs) on immune cells that facilitate the internalization of *Candida* cells by phagocytes (Rubin-Bejerano et al., 2007; Hall et al., 2013). This process may end up with the successful killing of the internalized fungal cells, or with a win by the fungus through proliferation and escape from the phagocytes, and subsequent further invasion of mucosal tissues.

This host–fungal interplay has been well documented, and several genes and pathways that are crucial for both phagocyte and fungal cell function have been characterized using genetic, microscopic and chemical approaches (Lorenz et al., 2004; Netea et al., 2006; McKenzie et al., 2010). An important characteristic of *C. albicans* influencing engulfment by phagocytic cells is its morphological state; morphology has been described as one the most crucial virulence features of this fungus (Saville et al., 2003; Hall, 2015). The commensal, typically yeast form cells of *C. albicans* seem generally to be compatible with host/fungal homeostasis and avoid triggering major induction of the host immune response. However, increased proliferation, and the switch to the hyphal state can alert the host immune response to initiate control over this microbial pathogen by phagocytic cells including neutrophils and macrophages (Lewis et al., 2012).

Although there is considerable information on this interface between the fungal cell and the phagocyte cell, many aspects of the *C. albicans* genetic circuits and molecular pathways required to manage the host response and control its own viability in the mammalian host are still incompletely understood. We conducted a screening of the interactions between a collection of *C. albicans* conditional mutants and cultured macrophages to investigate the kinetics of this fungal-macrophage cell interface and identify new *C. albicans* genes that may be involved in the interactions with macrophages. From our initial screening, we saw that the engulfment process of macrophage for *C. albicans* depends on fungal morphology, with yeast-locked mutants less engulfed than mutants that morphologically behaved as the wild-type, and with filamentation-locked mutants more highly engulfed. Furthermore, we identified, among those mutants that show a morphological pattern equivalent to wild-type, a number of candidates that showed modified kinetics of recognition and engulfment by macrophages. This will expand our knowledge of this opportunistic pathogenic fungus and provide new insights into the host immune response against *C. albicans.*

## Materials and Methods

### Genetic Screening of the *Candida albicans*-Macrophage Interactions

#### GRACE™ Library Growth

The GRACE™ (gene replacement and conditional expression) library (Roemer et al., 2003), was used as a source of *C. albicans* genes to screen for modifications in the interaction of the fungal cells with macrophages. This collection has about 2500 diploid strains, which were engineered by replacing the native promoter of one allele of each manipulated gene with a TET (tetracycline)-regulated-promoter and deleting the other copy of the gene, replacing it with an auxotrophic marker. Each modified gene can thus be conditionally repressed when cells are placed are under TET-promoter repression (Nakayama et al., 2000). Briefly, the library was streaked from frozen stocks on YPD agar plates (2% agar, 2% peptone, 1% yeast extract, 2% dextrose) supplemented with 50 mg/l uridine. Then, individual colonies for each mutant were grown in a pre-culture in liquid YPD media supplemented with uridine O/N at 30^°^C with 220 rpm shaking. Subsequently, an aliquot was measured at OD_600_ and a dilution to an OD_600_ of 0.3 was re-inoculated in 5ml of YPD uridine media containing 20 μg/ml of doxycycline and grown O/N at 30^°^C at 220 rpm, then diluted again to OD_600_ of 0.3 and grown for a further 3 h in YPD uridine with 20 μg/ml of doxycycline. Finally, cells were washed, counted and diluted for assessment for macrophage interaction.

### Macrophage Engulfment Assays

#### Murine Macrophages

The RAW 264.7 murine macrophage cell line was kindly provided by Dr. Albert Descoteaux (INRS-Armand-Frappier, Laval, QC, Canada). Macrophages were cultured in DMEM medium supplemented with 10% FBS, penicillin/streptomycin and HEPES. Macrophages were seeded at a 2 × 10^5^ density and grown for 48 h at 37°C and 5% CO_2._ Once cells reached 80% confluency, macrophages were collected with Trypsin-EDTA and then centrifuged for 10 min at 10,000 × rpm, at room temperature. Cells were then stained with trypan blue and counted, and an aliquot of 1.2 × 10^6^ viable cells/ml was prepared for further macrophage engulfment assays with the fungal cells.

#### Human Monocyte-Derived Macrophages

The protocol was approved by the Concordia University Human Research Ethics committee (certificate 30009292) and signed consent was obtained prior to the study. Venous blood was drawn from a human participant into ten heparinized vacutainer tubes. The participant was healthy as assessed by self-reporting of their health condition. In brief, the peripheral blood mononuclear cells (PBMC) were obtained using Ficoll-Hypaque density centrifugation techniques as previously described (Tabatabaei Shafiei et al., 2014). Monocytes were obtained using EasySep™ human CD14 positive selection kit according to manufacturer’s instructions. Subsequently, the PBMCs were cultured and differentiated to macrophages as described (Leascoat et al., 2018). Briefly, monocytes were cultured and differentiated into macrophages for 6 days using M-CSF (50 ng/ml) in RPMI medium, supplemented with 10% heat-inactivated calf bovine serum (CBS) and pen/strep. At day 6, differentiated macrophages were collected and counted for further engulfment assays testing *C. albicans* cells.

### High-Throughput Imaging of Macrophage Engulfment Kinetics of GRACE Mutants

#### Macrophage Engulfment Assays of Fungal Cells

An aliquot of each GRACE mutant and the parental strain (CAI4), was spun down, the media removed, and the cells washed three times with PBS (phosphate buffer saline). Then the fungal cell concentration was adjusted to 1 × 10^8^ cells/ml (Tóth et al., 2004). Next, fungal cells were stained with 50 mg/ml of Calcofluor White (CFW) and incubated for 10 min at RT (Williams and Lewis, 2011). The fungal cells were then washed three times in PBS, and finally a 1/100 dilution in PBS for each strain was prepared for the assay. For the initial screening, both macrophage and fungal cells of each strain were mixed together at a ratio 1:4 (*Candida* cells: macrophage), in wells of a 96-well plate and then visualized with the high-content screening microscope ImageXpress *XSL* wide-field (Molecular Devices, Sunnyvale, CA, United States). The 96 well plate was placed in a chamber equilibrated at 37°C and 5% CO_2_. Images were captured at 40X objective magnification on two channels (transmitted light, and DAPI). Every live-imaging interaction between macrophage and fungal cells was set to take images every 5 min during a 4 h total running time. Subsequently a time-lapse video was generated using the MetaXpress high content imaging acquisition and analysis software (Version 6.1.1, Molecular Devices). At least 300 macrophage per wide field (out of 6 wide fields per assay) were counted, including those with and without fungal cells internalized over the entire 4 h of the assay. Fungal cells (either budding, germ-tube forming or filament forms) engulfed by macrophages were counted and averaged for every six time-points corresponding to 30 min or 0.5 h as expressed in the figures, and normalized to the ratio: [number of fungal cells at time point X/number of fungal cells for every time point]/10 macrophages to generate an engulfment index (EI) (Tóth et al., 2004). The average macrophage uptake was calculated as the ratio of macrophages with engulfed fungal cells (stained or fluorescence signal) divided by the number of macrophages without fungal engulfment for 12 time points corresponding to 1 h of interaction, counted over six wide-field images, from three independent assays. Further time-lapse video data were edited using image-J software. Macrophage engulfment kinetic curves, histograms and statistical analysis were analyzed with GraphPad Prism (Version 6.0).

### Engineered *Candida albicans* Strains

From the initial screening of the *Candida*-macrophage interaction, we identified strains that showed a lower rate of engulfment compared with wild-type strain although they underwent an apparently normal yeast-hyphal transition. Selected genes were deleted by the transient CRISPR-Cas9 method applied in *C. albicans* (Min et al., 2016; see Table 1 about the strains used or generated in this study). These deletions were made in a CAI4 strain tagged with GFP (Marcil et al., 2002). Each component for CRISPR was independently amplified from the pV1093 plasmid (Vyas et al., 2015). For the repair template, the SAT-1 cassette, containing the nourseothricin (NAT) gene was engineered by fusion PCR, adding approximately 300 bp of the flanking sequence of the gene of interest upstream and downstream of NAT to direct gene replacement of the gene of interest Table 2 (primers description). The single-guide RNA (sgRNA) cassette was also generated from the same pV1093 plasmid. Both sequence specific and chimeric primers for fusion PCR were designed as follows (Vyas et al., 2015) (see Table 2 for details on each specific sgRNA for each deleted gene). A first round of PCR was run in order to generate two amplicons: The first one included a forward oligo, starting at a 800 bp up in the *SNR52* promoter was used with a reverse primer containing a specific 20 bp RNA guide, either targeting the *OPY1* or *KRE1* gene, followed by the end of the *SNR52* promoter. The second amplicon of 800 bp was amplified with a forward primer beginning with a sgRNA scaffold for each targeted gene, followed by the end of the *ENO1* terminator, and a reverse primer including the downstream *ENO1* terminator. A second step for extension of the chimeric primers was run in order to fuse both PCR products (Vyas et al., 2015). Finally, a nested PCR amplification with an outer upstream *SRN52* forward primer and a downstream reverse primer from the *ENO1* terminator, generated a nearly 1425 bp sgRNA cassette (Huang et al., 2021).

**TABLE 1:** *Candida albicans* strains used or generated in this study.

Strain	Background/Genotype	Author
CAI4 (*C. albicans* wild-type)	*ura3*::imm434/*ura3*::imm434	Fonzi and Irwin, 1993
CAI4-GFP (green fluorescence-protein tag)	*ura3*::imm434/*ura3*::imm434	Marcil et al., 2002
CAI4-RFP (red fluorescence-protein tag)	*ura3*::imm434/*ura3*::imm434	This study
Δ/Δ*opy1*-GFP	*ura3*::imm434/*ura3*::imm434:opy1	This study
Δ/Δ*kre1*-GFP	*ura3*::imm434/*ura3*::imm434:kre1	This study

**TABLE 2:** Primers used for RFP-tagged construct on CAI4 cells and for CRISPR-Cas 9 method.

Mlu1-RFP-gene/Fw*	5′AGAATacgcgtATAATGGTTTCAAAAGGTGAAGAAGATAATATG3′
Nhe1-RFP-gene/Rv*	5′AGACTAgctagcTTATTTATATAATTCATCCATACCACCAG3′
CaCas9/Fw	5′ATCTCATTAGATTTGGAACTTGTGGGTT3′
CaCas9/Rv	5′TTCGAGCGTCCCAAAACCTTCT3′
NAT Fw1	5′TTAGGCGTCATCCTGTGC3′
NAT Rv1	5′AAGAAAGAAAGAAAACCAGGAGTGAA3′
2SNR52/Fw	5′AGTATGACTACTATATCACAGTTTTAGAGCTAGAAATAGCAAGTTAAA3′
2SNR52/Rv	5′CGATAACTAAAGCAGCAACTTCTT3′
sgRNA/Nat/Fw	5′GCAGCTCAGTGATTAAGAGTAAAGATGG3′
sgRNA/Nat/Rv	5′ACAAATATTTAAACTCGGGACCTGG3′
sgRNA273/R_KRE1/Fw	5′GCGGCCGCAAGTGATTAGACT3′
sgRNA424/F_KRE1/Rv	5′TGTGATATAGTAGTCATACTCAAATTAAAAATAGTTTACGCAAGTC3′
KRE1UP/Fw2	5′AAATCATTAGGGAAATAGGAAAAGATATAAACGATTGGAATTTG TCCTTCTTCTAAAACC3′
OVNAT/KRE1-Dwn/Fw5	5′AAGCAGCAACTTCTTACTAACTGTTTCCCAGCTTCAAACAATCC3′
OVNAT/KRE1-Up/Rv2	5′GGAGCACAGGATGACGCCTAAGATGTGAAAGGTGTATTG3′
KREDw/NATOV/Rv5	5′TTTAGATTGACGTGTAAACCCCCAATGGACAACCAGCC3′
OPY1-NAT/Fw1:	5′CAGGCTCAAGTGAGGAGATCAACTTCTTTGACTG3′
NAT-OPY1/Rv1	5′GGCTAGATCGTTCCATGTTGAGTTGAGTTCAATCTTC3′
sgRNA279-298/OPY1/Fw	5′GAGTTATTATAAAGACTCCA3′
sgRNA443-298/OPY1/Rv	5′GGTGGATGGGAAGACACAGA3′
OPY1-UP/Fw	5CACCTTACAATGCTGTGATACGC3′
OV-OPY1/NAT-OPY1-Dwn/Rv	5′CTACACAATACATATATAAAACTCTGTAAATTTACTTAAAGAAGTTGCTGCTTATCGATAACG3′
OPY1-NAT/Fw	5′TTGTTATCATAAATTAAGCTTTTAGTGTTAATTTTAGTTTGAGATTTACAATCTTAGAATCAAATTAGGCGTCATCATCCTGTGC
	TCCGAGAACCAG3′
OPY1/Dwn-Rv	5′ATTGTAGATTAATTCATATATCGTTTTTCTACTACTATC3′

*Lower case indicates restriction sites added.

Transformation was carried out as described (Walther and Wendland, 2003) and modified cells were selected on YPD-NAT plates (200 μg/ml). Subsequently, resistant colonies were screened by colony PCR for NAT insertion at the desired locus to confirm gene deletion and replacement by the resistance gene marker. We made a separate construct in the CAI4 background strain by adding an RFP-tag as follows: primers including Mlu1 forward and Nhe1 reverse restriction sites (see list of oligos on Table 2) were designed to amplify a 711 bp mCherry (RFP) gene from plasmid pHES262 (pADH1-Msn2-mCherry) (Stewart-Ornstein et al., 2017). Further, a CIP10Act-cyc plasmid (Murad et al., 2000) was digested with Mlu1/Nhe1 restriction enzymes and the mCherry (RFP) fragment was ligated into the plasmid. Subsequently the RFP-expressing plasmid was transformed into CAI4 cells at the RP10 locus and the red fluorescent signal was verified by microscopy.

### Imaging of Macrophage Engulfment Kinetics for Selected *Candida albicans* Mutants

Detailed kinetics of macrophage and specific *C. albicans* knock-out strains were measured using a Nikon Eclipse TiE inverted epifluorescence microscope, including bright field illumination and fluorescent filters GFP/Alexa 488 and Cy3/Alexa 546/555/568, with cell growth chambers supplied with 5% CO_2_ and held at 37^°^C. Macrophages and GFP-tagged *C. albicans* mutants, in the presence or not of WT *C. albicans* tagged with RFP, were grown in the same conditions and prepared for visualization of the interactions as previously described. A 4 h long imaging profile was recorded for the interaction between macrophage and fungal cells. Then the kinetics of this interaction were analyzed under the same parameters used for the high-throughput imaging analysis.

## Results

### Screening *Candida albicans* GRACE Library Mutants for Interaction With Macrophages

**FIGURE 1:**
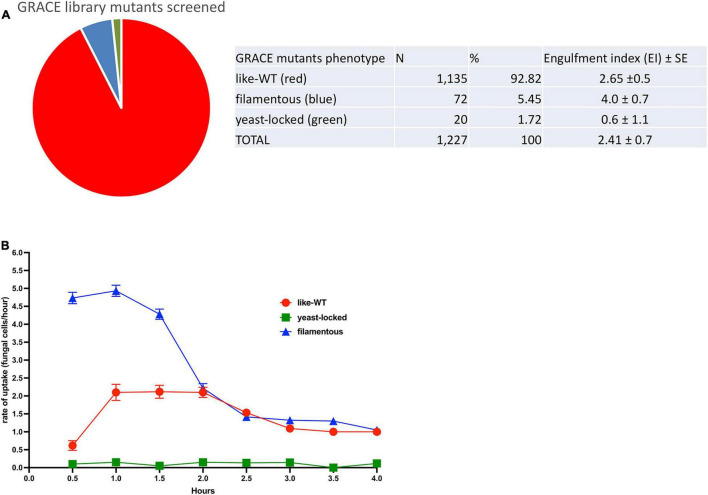
GRACE mutants-macrophage engulfment screening. **(A)** A total of 1227 mutants were screened for their interaction with macrophages. Three distinct groups were identified in this analysis that correlate with the fungal cell morphology. The main group found corresponds to strains like-wild-type where the mutation did not perturb the normal serum-induced morphological transition (92.82%), followed by filamentous mutants (5.45%) and yeast-locked mutants (1.72%). The overall engulfment rate observed by 10 macrophages/h was estimated for each mutant group: yeast-locked cells showed the lower engulfment rate (0.60 ± 1.1), followed by like-WT (wild-type) mutants (2.65 ± 0.5) and the filamentous mutants with the higher rate (4.0 ± 0.7). Average ± SE. **(B)** Kinetics of engulfment by murine macrophages, show a distinctive path for each phenotypic mutant group. Filamentous mutants (blue line) showed a higher engulfment kinetics over the first 2 h compared with the trace driven by the like-WT mutants (red line), which does an increasing trace from 0.5 to 1 h and then continued steady up to 2 h. After this time, the kinetic of both mutant groups, declined progressively up to the 4 h of the assay. In contrast the kinetic of mutants corresponding to the yeast-locked shape (green line), remained equally flat for over the entire 4 h of interaction between macrophage and fungal cells, indicating an almost absent recognition and internalization by the macrophages.

For the initial screening of the kinetics of interaction between *C. albicans* cells and macrophages, 1,227 strains from the GRACE library (Figure 1A) were investigated for their interaction with cultured mouse macrophage cells (see Supplementary Table 1). We identified three defined groups of *C. albicans* mutants based on morphology; the yeast-locked mutants that typically remained non-proliferating for the entire assay; a large group of mutants termed like-wild-type (like-WT) mutants that develop as normal Candida cells, initiating germ-tube formation with normal kinetics; and a third group corresponding to mutants with a constitutive filamentous phenotype that continue growing and extending their hyphal structures. Most of the mutants cultured from the GRACE library correspond to like-WT (93%), then a smaller group of 72 constitutive filamentous mutants (5.4%) as well as two dozen yeast-locked mutants that accounted for 1.6% of the total number of mutants screened. The overall average engulfment index over the 4 h interaction period shows clear differences related to the morphology phenotype of the GRACE mutants. The yeast-locked mutants were poorly recognised by the macrophages, reaching just an average engulfment index (EI), established over the entire 4 h of interaction, of 0.60 ± 1.1; this was followed by the like-WT mutants with an EI of 2.65 ± 0.5, and with the filamentous mutants showing the highest rate (EI of 4.0 ± 0.7) over this period (Figure 1A).

These differences are confirmed in the kinetics of interaction between specific *C. albicans* mutants and macrophages, monitored at specific timepoints (Figure 1B). At the early timepoints, the rates of engulfment by macrophages for the GRACE mutants are markedly different for each phenotypic group. Over the first 1.5 h of interaction, there was a steady and consistently higher rate of engulfment for the filamentous mutants. By contrast the like-WT mutants displayed an increasing rate of engulfment up to 1 h and then reached a continuous plateau. After 2 h of interaction, for both filamentous and like-WT mutants, the rate of engulfment by the macrophages tend to drop and to do so progressively until the end of the assay. We also noticed from our observations throughout the time-lapse videos that after 3 h of interaction a noticeable fraction of the fungal cells of both filamentous and like-WT mutants were growing out from the macrophages, either breaking out or dramatically stretching the macrophage membrane. In the case of filamentous strains, our observations tracking the recognition and engulfment of macrophages suggested that macrophages were ineffective at engulfing the tips of the filamentous mutants and arresting their growth (see representative Supplementary Video 3).

By contrast, the kinetics of engulfment for the yeast-locked mutants did not vary for the entire 4 h of incubation, and the macrophages barely engulfed these *Candida* yeast-cells. Many of these yeast-locked mutants correspond to essential genes encoding a variety of functions in *C. albicans* (see Supplementary Table 1). As many of these null mutants are involved in transcription, translation and chromosome segregation, it is likely that many cellular functions are absent in these mutants, interfering with development as a normal fungal cell, and affecting the production of surface antigens and fungal-derived molecules important in triggering macrophage engulfment.

**FIGURE 2:**
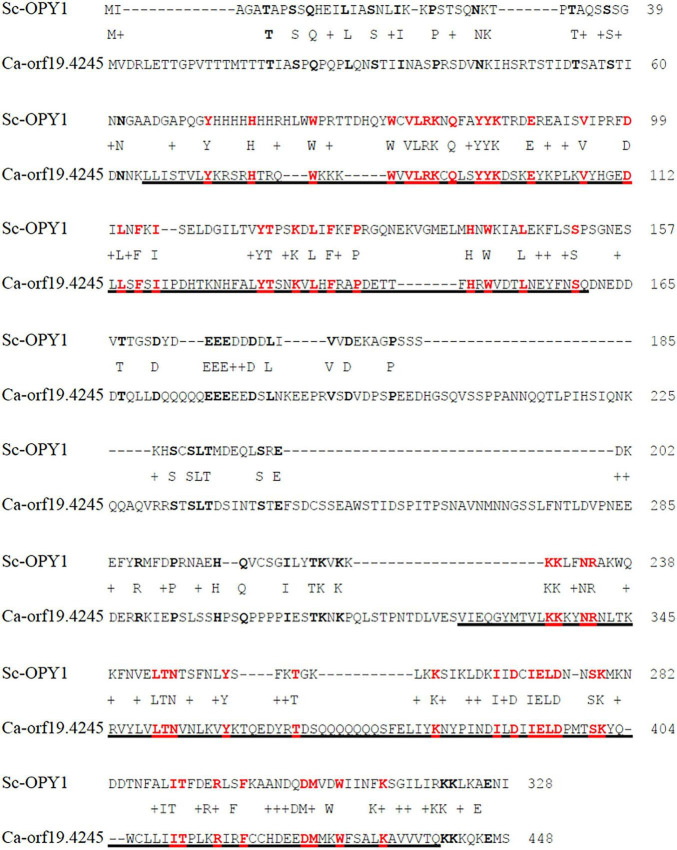
Pairwise protein alignment between *S. cerevisiae* Opy1 (Sc-Opy1) and *C. albicans* Orf19.4245. Amino acid identity was found to be 20%. Underlined sequences correspond to predicted Pleckstrin homology (PH) domains in Ca-Orf19.4245. Identities are highlighted with bold letters; identities within the PH domains are bold in red. Alignment made with Global alignment software, available on BLAST ^®^ from the NCBI website (https://blast.ncbi.nlm.nih.gov/Blast.cgi#). Protein sequence scanned and predicted with SMART bioinformatics software (http://smart.embl.de).

Overall, most of the mutants screened showed similar kinetics to the CAI4-wild-type strain (tagged in red) for interaction with and internalization by the macrophages. However, in the group of like-WT mutants we found a few strains that had a morphological development (from budding yeast, germ tube formation and hyphal development) similar to the wild-type, but which showed a clearly reduced rate of engulfment by the macrophages (see Table 3). Two of the mutated genes in the strains identified in this class were the ortholog of the *Saccharomyces cerevisiae* gene *KRE1* (Killer toxin REsistant 1), and an uncharacterized gene *ORF19.4245*. We examined this uncharacterized gene in more detail. A BLAST alignment search and further structural assessment identified this candidate as the likely ortholog of the *S. cerevisiae* gene *OPY1*. This identification was based on the structural alignment analysis, which showed similarly positioned and conserved Pleckstrin-homology (PH) domains in both the *C. albicans* and *S. cerevisiae* proteins (Figure 2). Functional analysis of the *OPY1* gene in *S. cerevisiae* suggests it plays a role in regulating the phosphatydilinositol-4,5-biphosphate [(PtdIns 4,5)P_2_] lipid content through interaction with the PtdIns(4)P 5-kinase, Mss4 (Ling et al., 2012).

**TABLE 3:** GRACE mutant hits with phenotype as like-WT, with reduced rate of macrophage engulfment compared with CAI4-WT, and their putative localisation on *C. albicans*, as described on the Candida Genome data base.

Gene name/ID	Function	% of engulfment rate compared to WT	Localization
TPS1	Trehalose-6-phosphate synthase; role in hyphal growth and virulence in mouse systemic infection; induced in presence of human neutrophils. Macrophage/pseudohyphal-repressed after 16 h; stationary phase enriched protein; Hap43-repressed Macrophage/pseudohyphal-repressed after 16 h; stationary phase enriched protein; Hap43-repressed	46%	Cytoplasm
UGP1	UTP-glucose-1-phosphatidyl transferase; localizes to yeast, not hyphal cell surface; Hog1-repressed. Stationary phase enriched; induced in oropharyngeal candidiasis; rat catheter biofilm repressed; Bcr1-repressed in RPMI a/a biofilms	46%	PM, CW
HAP43/CAP2	CCAAT-binding factor-dependent transcription factor; repressor; required for low iron response; similar to bZIP transcription factor AP-1; repressed by Sfu1; ciclopirox olamine induced; rat catheter, Spider biofilm induced	46%	Nucleus
RPC11	Putative RNA polymerase III subunit C11; repressed in core caspofungin response; Spider biofilm induced	45%	Cytoplasm
VPS4	AAA-ATPase involved in transport from MVB to the vacuole and ESCRT-III complex disassembly; mutation decreases SAP secretion and virulence in murine intravenous infection; regulated by Gcn2p, Gcn4p; required for normal Rim8p processingGcn4p; required for normal Rim8p processing	43%	Cytoplasm/vesicle biogenesis
ALG13	Ortholog(s) have *N*-acetyl glucoseaminyl diphosphodolichol *N*-acetylglucosaminyl transferase activity and role in dolichol-linked oligosaccharide biosynthetic process	41%	Cytoplasm/ER
PGA4	GPI-anchored cell surface protein; beta-1,3-glucanosyltransferase with similarity to the *A. fumigatus* GEL Family transcript induced in RHE model of oral candidiasis; fluconazol-induced	40%	CWP
NAT2	Putative N-terminal acetyltransferase; Hap43p-repressed gene; mutation confers hypersensitivity to toxic ergosterol analog	39%	Cytoplasm
GCD7	Putative translation initiator; down regulated in the presence of human whole blood or polymorphonuclear (PMN) cells	38%	Cytoplasm
GCD2	Putative translation initiation factor; genes encoding ribosomal subunits, translation factors, and tRNA synthetases are downregulated upon phagocytosis	38%	Cytoplasm
orf19.1285	Plasma membrane-localized protein of unknown function; Hap43p-repressed gene	37%	PM
VMA5	Putative vacuolar H(+)-ATPase; plasma membrane localized; rat catheter biofilm repressed	35%	PM
CAN1	Basic amino acid permease; arginine metabolism; regulated by Nrg1/Tup1; caspofungin, flucytosine induced; colony morphology-related regulation by Ssn6; Hap43-repressed; rat catheter and Spider biofilm induced; promoter bound by Efg1	34%	PM
PHR2	Glycosidase; role in vaginal not systemic infection (low pH not neutral); low pH, high iron, fluconazole, Hap43-induced; Rim101-repressed at pH8; rat catheter biofilm induced; Bcr1-repressed in RPMI a/a biofilms	22%	PM/mitochondria
OPY1	Ortholog of *S. cerevisiae* ortholog Overproduction-induced Pheromone-resistant Yeast	14%	PM
KRE1	Cell wall glycoprotein; beta glucan synthesis; increases glucan content in *S. cerevisiae* kre1, complements killer toxin sensitivity; caspofungin induced; Spider/rat catheter/flow model biofilm induced; Bcr1-repressed in RPMI a/a biofilms	6.7%	CW

*CW, cell wall; ER, endoplasmic reticulum; PM, plasma membrane.*

**FIGURE 3:**
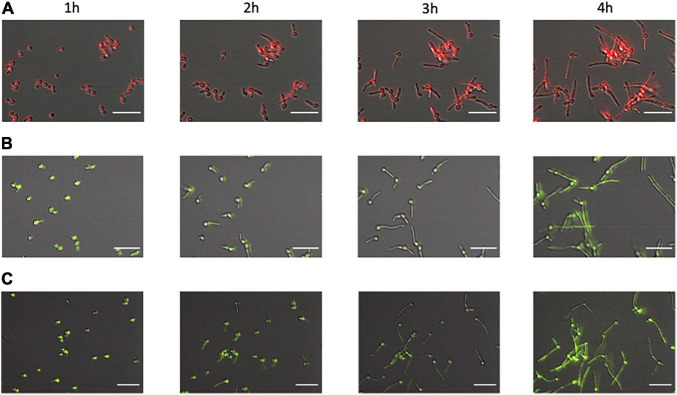
Development of the C albicans morphological phenotype over time. We compared fungal cells from WT-RFP **(A)**, Δ/Δ*opy1*-GFP **(B),** and Δ/Δ*kre1*-GFP **(C)**. All cells, regardless of phenotype or tag, showed the same morphological pattern. At 1 h of incubation most of them are still in the budding form with a few showing germ tube initiations. At 2 h there are frequent germ tubes growing from the original yeast cells. At 3 and 4 h there are extended filamentous cells and frequent initiations of a second filament from an original yeast cell. Scale bar = 20 μm.

Following the establishment at the differences in the kinetics of engulfment between these two *Candida* mutants and WT-RFP, we tested if the presence of phagocytes could produce any differences in the growth and development of either the knock-out or the parental strains. We separately incubated each of the Δ/Δ *kre1*-GFP, Δ/Δ*opy1*-GFP and WT-RFP strains in the fungal-macrophage medium; all strains developed normally from yeast, through germ tube formation and finally into pseudo-hyphae/filamentous fungal cells (Figure 3). When analyzed in detail, we did not find differences in the growth and normal development of the fungal cells in the presence or absence of the murine phagocytes.

**FIGURE 4:**
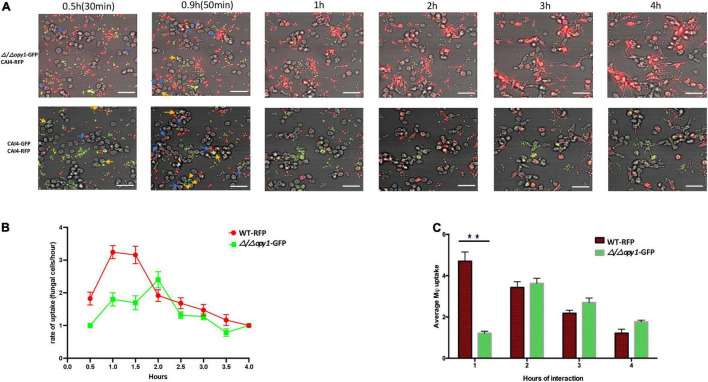
Kinetics of engulfment by macrophages of fluorescently-tagged *C. albicans* cells, with red (WT-RFP) and green tagged cells (Δ/Δ*/opy1*-GFP mutant) incubated together. RAW 264.7 macrophages were incubated for 4 h with *C. albicans* wild-type and mutants. **(A)** Microscopy images depicting the kinetics of macrophage engulfment of red-fluorescing CAI-4-RFP WT cells and green-fluorescing Δ/Δ*opy1*-GFP mutant cells. Blue arrows indicate WT-RFP cells engulfed by macrophages and yellow arrows indicate (Δ/Δ*opy1*-GFP mutant cells engulfed by macrophages. **(B)** The mutant *(Δ/Δ/opy1*-GFP) showed a delayed engulfment compared with the WT strain. **(C)** This is reflected in the average engulfment rate every hour of interaction when compared with the WT-RFP values; the mutant shows significantly reduced engulfment at the 1 h time point, Blue arrows indicate fungal cell engulfment by macrophages at different time points. Statistical test by two-way ANOVA. **p-value > 0.001. Scale bar = 20 μm.

We constructed non-conditional null mutant strains Δ/Δ*opy1* and Δ/Δ*kre1* and assessed their kinetics of engulfment by macrophages; these strains confirmed the different pattern of engulfment relative to the wild-type. Figure 4 shows a direct comparison between the wild-type-CAI4-RFP *C. albicans* cells (WT-RFP) and the GFP tagged Δ/Δ*opy1* mutant. We observed that macrophages recognized and internalized fungal cells at early time points (0.5 h–0.9 h), with a marked preference for WT-RFP compared with Δ/Δ*opy1* mutant (Figure 4A, top panel). This trend on the preference of WT-RFP cells over the Δ/Δ*opy1* mutant by the macrophages changed throughout the imaging of the fungal cell-macrophage interaction (Figure 4B). The kinetics clearly showed a lower engulfment rate on the Δ/Δ*opy1* mutant at the initial time points (0.5, 0.9, 1 h) and then, coinciding with germ tube formation, the engulfment for Δ/Δ*opy1* mutant increased slightly over the WT-RFP cells, and finally both strains exhibit similar kinetics in the recognition and internalization by the macrophages (Figures 4B,C). We also imaged the same assay including WT-RFP and the background WT-GFP (Figure 4A lower panel). For these controls, the kinetics of engulfment by the macrophages are similar, and display a few initial internalizations by the macrophages (0.5, 0.9 h) and then continuous engulfment kinetics as seen for the WT-RFP and Δ/Δ*opy1.*

**FIGURE 5:**
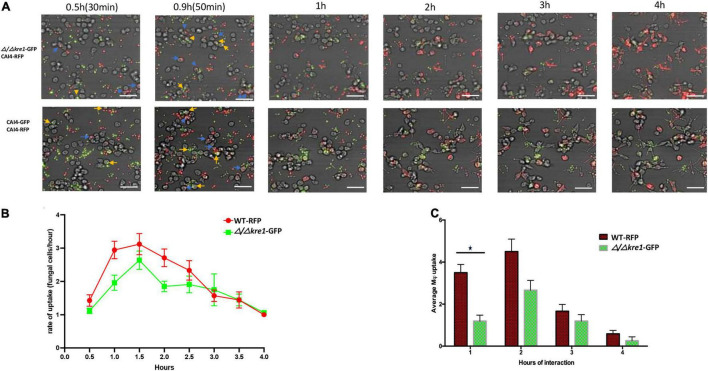
Kinetics of engulfment by macrophages of fluorescently-tagged *C. albicans* cells, with red (WT-RFP) and green tagged cells (Δ/Δ*kre1*-GFP mutant) incubated together. RAW 264.7 macrophages were incubated for 4 h with *C. albicans* wild-type and mutants. **(A)** Microscopy images depicting the kinetics of macrophage engulfment of red-fluorescing CAI-4-RFP WT cells and green-fluorescing Δ/Δ*kre1*-GFP mutant cells. Blue arrows indicate WT-RFP cells engulfed by macrophages and yellow arrows indicate Δ/Δ*kre1*-GFP mutant cells engulfed by macrophages. **(B)** The mutant *(Δ/Δkre*-GFP) showed reduced kinetics of engulfment compared with the WT strain. **(C)** This is reflected by reduced rates at all time points when compared with the WT-RFP values. Blue arrows indicate fungal cell engulfment by macrophages at different time points. Statistical test by two-way ANOVA. *p-value > 0.005. Scale bar = 20 μm.

We performed the same direct comparison assay for the GFP tagged Δ/Δ*kre1* mutant and WT-RFP cells in their interaction with the macrophages (Figures 5A–C). We also found an early and clear preference of the macrophages to engulf WT-RFP cells over Δ/Δ*kre1*-GFP mutants (Figure 5A top panel). In this case the kinetics of engulfment by the macrophages remained biased to the WT-RFP Candida cells compared with the Δ/Δ*opy11*-GFP mutants (Figure 5B) for the entire 4 h of imaging assay. This was supported with the average uptake by macrophages of fungal cells, with a non-significant but slightly higher rate for the WT-RFP over *the Δ/Δ kre1*-GFP mutants (Figure 5C). In comparison with the assay including both CAI4-RFP and CAI4-GFP (Figure 5A lower panel), the macrophages recognized and internalized both WT-tagged cells with no significant preference as seen previously (Figure 5A lower panel), and the engulfment kinetics remained equivalent through the 4 h of interaction.

We also tested the kinetics of macrophage engulfment when incubating either mutants or WT-RFP with the macrophages separately (see Supplementary Figures 1, 2). In both cases, the engulfment patterns of the mutants were very similar to that seen in the mixed cultures. The Δ/Δ*opy1*-GFP mutants showed a delayed recognition and engulfment by the macrophages, most dramatically at the initial time point of 0.5 h; engulfment then increased up to the 2 h point of interaction (Supplementary Figure 1B) and then decreased gradually until 4 h. By contrast, the WT-RFP engulfment pattern showed a higher rate of engulfment over the first 1 h of interaction and then dropped steadily down until the end of the 4 h of interaction. The average fungal cell uptake by macrophages per hour also shows greater internalization of WT-RFP cells compared with the Δ/Δ*opy1*-GFP mutant, which later increased after the 2 h up to the end of the 4 h of interaction (Supplementary Figure 1C), behaving similarly to the kinetics of internalization (Supplementary Figure 1B). The Δ/Δ *kre1*-GFP mutants incubated separately from the WT-RFP cells also showed a pattern of engulfment by the mouse macrophages similar to that in the mixed cultures. In this case both Δ/Δ *kre1*-GFP and WT-RFP cells had similar patterns of engulfment through the monitored period, however the WT-RFP always displayed a higher rate compared with the Δ/Δ *kre1*-GFP mutants at each time point (Supplementary Figures 2A,B). At 2.5 h the kinetics of engulfment change due to a drop in macrophage internalization, and then continue decreasing until 4 h of interaction. We also observed that after 3 h of the interaction, many fungal cells once engulfed by the macrophages were able to extend out through the phagocytes once they initiated filamentation. Overall, the average uptake by the macrophages correlates with the kinetics, showing a higher rate-for the WT-RFPs compared with the Δ/Δ *kre1*-GFP mutants over the 4 h interaction assay (Supplementary Figure 2C).

**FIGURE 6:**
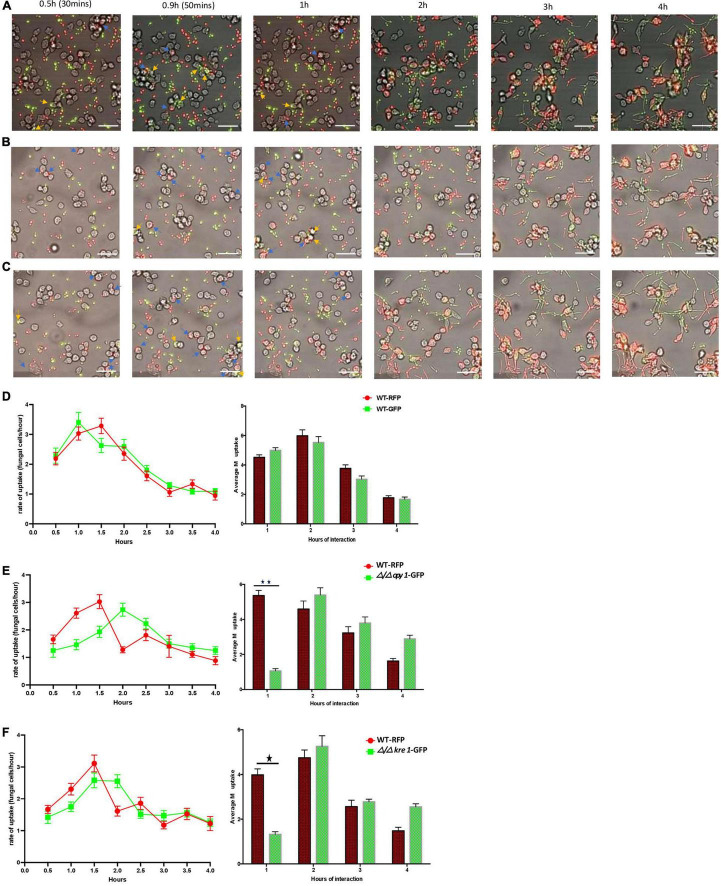
Kinetics of engulfment by macrophages of *C. albicans* (WT and mutants) incubated together with human monocyte-derived macrophages. **(A)** Kinetics of engulfment of CAI-4 WT cells tagged with GFP (green) or RFP (red). Compared with the WT strain [either RFP or RFP tagged on **(D)**], both mutants [Δ/Δ*opy1*-GFP on **(E)**] and Δ/Δ*kre1*-GFP **(F)** produced distinct and reduced engulfment kinetics, different macrophage uptake, specially marked at 1h of interaction. Blue arrows indicate WT-RFP cells and yellow engulfed of WT-GFP by macrophages. **(B)** Kinetics of engulfment displayed by macrophages for Δ/Δ*opy1*-GFP (green) and WT-RFP (red). Blue arrows indicate WT-RFP cells engulfed by macrophages and yellow arrows indicate Δ/Δ*opy1*-GFP mutant cells engulfed by macrophages **(C)**. The engulfment kinetics by macrophages of Δ/Δ*kre1*-GFP (green) and WT-RFP (red). Both mutants produced distinct and reduced engulfment kinetics compared with the WT strain. Blue arrows indicate WT-RFP cells engulfed by macrophages and yellow arrows indicate Δ/Δ*kre1*-GFP mutant cells engulfed by macrophages. This is reflected as well in the average engulfment rate every hour of interaction when they are compared with the WT-RFP values. Statistical test by two-way ANOVA. *p-value > 0.005. ^**^p-value > 0.001. With this add, both *p*-values are incorporated on the legend and makes it clear and consistent with the histograms on **(E)** and **(F)**. Scale bar = 20 μm.

We initially used murine RAW264.7 macrophages to study the *C. albicans* GRACE library of conditional mutants and to investigate in detail two hits, *KRE1* and the uncharacterized *ORF19.4245*, identified as the ortholog of *S. cerevisiae OPY1* from the original screening. To further investigate these fungal-macrophage interactions we repeated the assay with both Δ/Δ *kre1*-GFP and Δ/Δ *opy1*-GFP using human monocyte-derived macrophages. We tested each GFP-tagged mutant in the presence of WT-RFP and the human monocyte-derived macrophages (Figure 6). In order to test the ability of the new phagocytic cell line and avoid any bias from the background CAI-4 we first incubated the monocytes with both WT-RFP and WT-GFP (Figure 6A). After the 4 h incubation assay, we found that both WTs produced similar kinetics of engulfment with increasing engulfment from 0.5 h reaching a peak between 1 and 1.5 h to then slowly decreasing to the 4 h time point (Figure 6D). When we tested the Δ/Δ *kre1*-GFP or the Δ/Δ *opy1*-GFP mutants along with WT-RFP cells and these human monocyte-derived macrophages, we saw different kinetics of engulfment for both mutants compared with the WT-RFP. Whereas the WT-RPF kinetics remain similar to the control pattern, increasing in the period 0.5–1.5 h and then dropping slowly until 4 h (Figures 6E,F), the kinetics for the mutants Δ/Δ *opy1*-GFP or Δ/Δ *kre1*-GFP exhibited reduced macrophage recognition compared with the WT-RFP. The most significant difference occurred at 2 h of the assay, when both mutants displayed a peak of macrophage engulfment, and coinciding with the drop of internalization of the WT-RFP, suggesting a delayed response. When we analysed the average macrophage uptake, again we found substantial differences between WT-RPF and Δ/Δ *opy1*-GFP (Figure 6E) and Δ/Δ *kre1*-GFP (Figure 6F) at 1h of incubation, but little difference after that point. Thus, it appears that both mutants reduce macrophage internalization relative to the WT-RFP cells.

## Discussion

How the pathogenic fungal microbe, *Candida albicans*, regulates its behaviour to face the immune response represented by phagocytes, is a matter that remains under intensive investigation. In the present study, we performed a genetic screen of *C. albicans* mutants and assessed their interaction with macrophages by monitoring the kinetics of this interaction from initial recognition to internalization of the fungal cells. Our screening of the GRACE library suggests that the interaction between *C. albicans* cells and macrophages relies significantly on the morphology of the fungal cells. There was a clear preference in the recognition and engulfment by macrophages of mutants with a filamentous phenotype. Less effectively engulfed were mutants that develop similar to WT cells, starting as budding cells, but initiating germ tube formation followed by hyphal extension in the serum-containing media growing the macrophage cells. Finally, cells that remained locked in the yeast form were poorly recognized and engulfed.

Our present observations are consistent with other studies (Kumamoto and Vinces, 2005; Lewis et al., 2012), and have extended the analysis to over 1,200 mutants from the GRACE library. Several lines of evidence suggest that aspects of the cell surface of hyphal cells are an important signal for macrophage recognition. First, hyphal-locked mutants are efficiently engulfed from the initiation of the interaction, while yeast-locked cells remain recalcitrant to engulfment. Second, the majority of the mutant cells that undergo the yeast-hyphal transition are more efficiently engulfed after the initiation of germ tube development. Finally, two mutants that reduce the efficiency of engulfment in cells that undergo a classic germ tube initiation and hyphal transition are implicated in aspects of cell wall and cell membrane formation.

In the group of budding-arrested mutants, the poor rate of macrophage engulfment is interesting, because many of these variants lack functions critical for fungal cells, such as translation initiation factors and elements required for chromosome segregation. It is possible that these budding-defective mutants are poorly recognized and internalized by macrophages, due to reduced cell growth. Alternatively, many of these yeast-locked mutants may be lacking the production of fungal-derived biomolecules such as farnesol (Ghosh et al., 2010), which have been linked to immune cell activation, pathogen recognition and internalization (Blasi et al., 1992; Ghosh et al., 2010; Mukaremera et al., 2017). These characteristics, perhaps, would make these mutants less visible to the macrophages.

In contrast, a rapid rate of engulfment was identified for those mutants with a constitutive filamentation phenotype when they were examined for their interaction with macrophages. In this group there are genes encoding proteins involved in the biogenesis of ribosomal subunits and translation factors, as well as various enzymes that regulate oxidative stress, ATP-hydrolysis and amino acid biosynthesis. The marked preference of macrophages for this group of mutants may have several explanations; in particular these filamentous cells may offer a wide set of cell wall antigens with the potential to be recognized by immune cells (Cassone et al., 1987; Urban et al., 2003; Shen et al., 2015). Typically, once macrophages have internalized pathogens, they activate the phagosome to trigger destruction of the engulfed cells (Bain et al., 2014), In our observations, macrophages were able to internalize hyper-filamentous strains from the GRACE library screened in our study as seen before (Marcil et al., 2002). We also noticed that some hyphal mutants produced elongated filaments in different planes and directions, making them inaccessible to complete engulfment by the macrophages (Supplementary Video 3).

At later timepoints (3 h) we observed that some of the WT *Candida* and the KO mutants were able to break through from macrophages at close to 10% of the engulfment events, compared with a less than 5% for the hyper-filamentous mutants screened in our study. This partial lack of killing capacity of macrophages when they face longer hyphal cells or elongated fungal cells inside them, relies on the inhibition in forming and maturing the phagosome (Bain et al., 2014; Maxson et al., 2018), the organelle responsible for inactivating microorganisms internalized by macrophages. Usually, *Candida* cells can escape from phagocytes by affecting inflammasome-dependent pyroptosis at early time points, based on the multiplicity of infection (MOI) (Wellington et al., 2014) and ultimately lysing the macrophage as the fungal cells continue to grow. Under our conditions, a MOI of 1:4 (*Candida*: macrophages), there was a delayed effect on the macrophage pyroptosis compared with previous publications that used higher number of *Candida* cells (MOI 10) (Wellington et al., 2014; Ding et al., 2021). Once fungal cells escape the phagocytes there will be an impact on the macrophage uptake capacity at later time points that may influence the macrophage uptake index, which decreases substantially in the last 2h of interaction in our assay, as visualized in Supplementary Videos 4–12.

In our tested portion of the GRACE library collection, we found that after gene repression almost 80% of the strains displayed the typical growth pattern of *C. albicans* WT cells. Even in the repressed state, these cells develop as morphologically normal, starting as yeast cells, then initiating germ tube formation and ultimately forming hyphal or pseudo-hyphal forms in the presence of macrophages. This group of strains were differentially recognized over the course of the morphological transitions; the budding forms were poorly recognized, while after germ tube initiation (after 50 min) and later filamentous extension, cells were efficiently internalized, reaching their maximum engulfment by macrophages between the first and second hour (Figure 1). However, within this class of cells we identified a set of repressed genes that generated, in their interface with macrophages, reduced engulfment kinetics compared with the WT (Table 3). Among these mutants, a few genes are involved in metabolic pathways (*TPS1*, *UGP1*, *ALG13*), transcription (*HAP43*/*CAP2*, *RCP11*), translation initiation (*GCD7*, *GCD2*), vesicle trafficking (*VPS4*), while many of the genes found with lower kinetics are linked to the plasma membrane and cell wall (Zaragoza et al., 1998; Lorenz et al., 2004; Bruno et al., 2006; Hall and Gow, 2013).

We have focused on two genes whose inactivation triggered reduced engulfment rates in the interaction with the macrophages with no evident impact on the morphological transition: *KRE1* and *OPY1*. We created null mutants by CRISPR gene editing and challenged these mutants in the fungal-phagocyte engulfment assay. We confirmed that, as previously observed from the GRACE library conditionally inactivated strains, both Δ/Δ *opy1* and Δ/Δ *kre1* mutants have a distinctive kinetics in the interaction with macrophages compared to the WT. When we investigated the fungal-macrophage kinetics during the first two hours of interaction when most of the engulfment occur, the Δ/Δ *kre1* mutant showed a reduced kinetics compared to the WT, but somewhat higher compared with our observation of the *KRE1*-GRACE mutant (data not shown). Kre1 (killer resistant 1) is part of a family of glycoproteins anchored in the cell wall, originally described in *Saccharomyces cerevisiae* (Breing et al., 2004), to be involved in β-1,6-glucan synthesis (Boone et al., 1990). Although the mutant Δ/Δ *kre1* may produce an altered β-1,6-glucan distribution, the sugar cell wall constitution in *C. albicans* has been described with a more abundant composition of β-1,3-glucan rather than β-1,6-glucan (Boone et al., 1991). It is possible that alteration of β-1,6-glucan synthesis at the cell wall in *C. albicans* may provide variable recognition by macrophages, because of the constant remodeling of this fungal cell structure enriched by other sugars and proteins that serve as pathogen-associated molecular patterns (PAMPS) (Klis et al., 2001; Hopke et al., 2016). Overall, when we tested the Δ/Δ *kre1*-GFP mutant with either murine or human-derived macrophages, there was a consistent decrease in the engulfment rate for this mutant, mainly in the first 2h of interaction, compared with the Candida WT-RFP cells (Figures 5, 6C,F and Supplementary Figures 1B, 2B).

The Δ/Δ*opy1* null mutant exhibited lower and delayed internalization by macrophages, also consistent with the GRACE library strain. This identifies a new gene, predicted to be involved in phospho-inositol metabolism, in macrophage engulfment of *C. albicans* cells. How this gene influences macrophage recognition is unclear. Its ortholog in *S. cerevisiae* (ScOpy1) has been described as regulating the phosphatydilinositol-4,5-biphosphate [(PtdIns 4,5)P_2_] lipid content (35) through direct binding with the kinase Mss4 to regulate PtdIns (4,5)P_2_, at the inner leaf of the plasma membrane in yeast. PtdIns (4,5)P_2_ represent signaling lipids that are implicated in eukaryotic cells in endocytosis, actin binding in the cytoskeleton and cell volume maintenance (Ene et al., 2015; Hammond, 2016). In addition, PtdIns (4,5)P_2_ are essential lipids that maintain plasma membrane components such as proteins that insert into the lipid bilayer and constitute the scaffold for the cell wall outer structure (Hammond, 2016). We observed at early time points in the fungal cell-macrophage interaction, when fungal cells were in the budding form, macrophages have a marked preference for *Candida* WT rather than the Δ/Δ*opy1* strain. The absence of Opy1 may alter the plasma membrane or may affect and modify the proper building and arrangement of cell wall components such as chitin and β-glucan-anchored proteins linked to glycophosphatidylinositol (GPI) (McLaughlin and Murray, 2005; Badrane et al., 2016). Over the middle part of the interaction (1–2 h) during germ tube extension and pseudohyphe growth, the kinetics of the recognition and engulfment of macrophages for Δ/Δ*opy1* mutants was delayed and reduced compared with *Candida* WT cells. This was seen by our wide-field microscopy visualization as well as in the automated counting generated in the cell sorter experiment. During the end of the assay (2–4 h), there was much less difference in the phagocyte’s internalization of filamentous Δ/Δ *opy1* and WT cells, suggesting the impact of the modulation in PtdIns (4,5)P_2_ is primarily limited to the earlier events.

This phenotype observed in Δ/Δ *opy1* mutants, was confirmed when we challenged these cells in presence of macrophages and a few *Candida* WT cells, and it was observed the predilection of the macrophage to take in the WT cells over the Δ/Δ *opy1* mutants. All this suggests that Opy1 function regulating PtdIns (4,5)P_2_ levels at the plasma membrane has a significant consequence for the initial recognition by macrophages and that phosphotidyl-inosositides function in the fungal-macrophage interplay. This conclusion it may have implications for our understanding of the *C albicans* structural arrangements that drive the immune response from phagocytes. This interaction may depend not just on the cell wall components and fungal released molecules (Richard and Plaine, 2007; Chaffin, 2008), but also on the addition of PtdIns (4,5)P_2_ lipids to ensure normal functioning on the outer layers of *C. albicans* allowing the pathogen cells to “be seen” and ingested by macrophages at early stages of *C. albicans* infection.

## Data Availability Statement

The original contributions presented in the study are included in the article/Supplementary Material, further inquiries can be directed to the corresponding authors.

## Ethics Statement

The studies involving human participants were reviewed and approved by Concordia University Human Research Ethics Committee. 7141 Sherbrooke Street West, H4B1R6, QC, Canada. The patients/participants provided their written informed consent to participate in this study.

## Author Contributions

MW and PG: rational, experimental design, and manuscript writing. PG and PD: experimental work. PG: data curation and analysis. All authors contributed to the article and approved the submitted version.

## Conflict of Interest

The authors declare that the research was conducted in the absence of any commercial or financial relationships that could be construed as a potential conflict of interest.

## Publisher’s Note

All claims expressed in this article are solely those of the authors and do not necessarily represent those of their affiliated organizations, or those of the publisher, the editors and the reviewers. Any product that may be evaluated in this article, or claim that may be made by its manufacturer, is not guaranteed or endorsed by the publisher.

## Supplementary Material

Supplementary Figure 1 The kinetics of engulfment displayed by macrophages of ΔΔ/*opy1*-GFP (green) and WT-RFP (red) separately.

Supplementary Figure 2 The kinetics of engulfment displayed by macrophages of ΔΔ/*kre1*-GFP (green) and WT-RFP (red) separately.

Supplementary Table 1 Of *C. albicans* GRACE conditional mutants screened in this study.

Supplementary Video 1 Video for GRACE library screening with the 274.RAW-macrophages 40X magnification. Representative of a yeast-locked mutant stained with Calcofluor (CW).

Supplementary Video 2 Video for GRACE library screening with the 274.RAW-macrophages 40X magnification. Representative of like-WT mutant stained with Calcofluor (CW).

Supplementary Video 3 Video for GRACE library screening with the 274.RAW-macrophages 40X magnification. Representative of a filamentous mutants stained with Calcofluor (CW).

Supplementary Video 4 Video for *OPY1* together with WT-RFP in presence of RAW267.4 macrophages. Time-lapse video (4 h) on macrophage/fungal cell interactions. Mutants Δ/Δ*opy1*-GFP or Δ/Δ*kre1*-GFP and WT-RFP in presence of 274.RAW-macrophages. 40X magnification.

Supplementary Video 5 Video for *KRE1* together with WT-RFP in presence of RAW267.4 macrophages. Time-lapse video (4 h) on macrophage/fungal cell interactions. Mutants Δ/Δ*opy1*-GFP or Δ/Δ*kre1*-GFP and WT-RFP in presence of 274.RAW-macrophages. 40X magnification.

Supplementary Video 6 Video for *OPY1* engulfment kinetics separated. Time-lapse video (4 h) on Macrophage/fungal cell interactions. Mutants Δ/Δ*opy1*-GFP or Δ/Δ*kre1*-GFP and WT-RFP in presence of 274.RAW-macrophages. 40X magnification.

Supplementary Video 7 Video for WT-RFP engulfment kinetics separated for comparison with (SV-6) Δ/Δ*opy1*-GFP. Time-lapse video (4 h) on macrophage/fungal cell interactions. CAI4 WT-RFP in presence of 274.RAW-macrophages. 40X magnification.

Supplementary Video 8 Video for *KRE1* engulfment kinetics separated. Time-lapse video (4 h) on macrophage/fungal cell interactions. Mutants Δ/Δ*opy1*-GFP or Δ/Δ*kre1*-GFP and WT-RFP in presence of 274.RAW-macrophages. 40X magnification.

Supplementary Video 9 Video for WT-RFP engulfment kinetics separated for comparison with (Supplementary Video 6) Δ/Δ*kre1*-GFP. Time-lapse video (4 h) on macrophage/fungal cell interactions. CAI4 WT-RFP in presence of 274.RAW-macrophages. 40X magnification.

Supplementary Video 10 Video for *OPY1* together with human monocyte-derived macrophages. Time-lapse videos (4 h) on Macrophage/fungal cell interactions: WT-RFP cells and mutant Δ/Δ*opy1*-GFP in presence of human-derived macrophages. 40X magnification.

Supplementary Video 11 Video for *KRE1* together with human monocyte-derived macrophages. Time-lapse videos (4 h) on Macrophage/fungal cell interactions: WT-RFP cells and mutant Δ/Δ*kre1*-GFP in presence of human-derived macrophages. 40X magnification.

Supplementary Video 12 Video for CAI4 background strains, WT-GFP and WT-RFP together with human monocyte-derived macrophages. Time-lapse videos (4 h) on Macrophage/fungal cell interactions. 40X magnification.

Supplementary Video 13 Video for non-stained WT-Candida and macrophages as control for the screening of Candida GRACE strains stained with Calcofluor White (CFW) and interaction with macrophages.
