# Supplementary figures and images for: Genetic Screening of Candida albicans Inactivation Mutants Identifies New Genes Involved in Macrophage-Fungal Cell Interactions
Source: Front Microbiol. 2022 Apr 5;13:833655. doi: 10.3389/fmicb.2022.833655 (PMC9016338; doi:10.3389/fmicb.2022.833655)

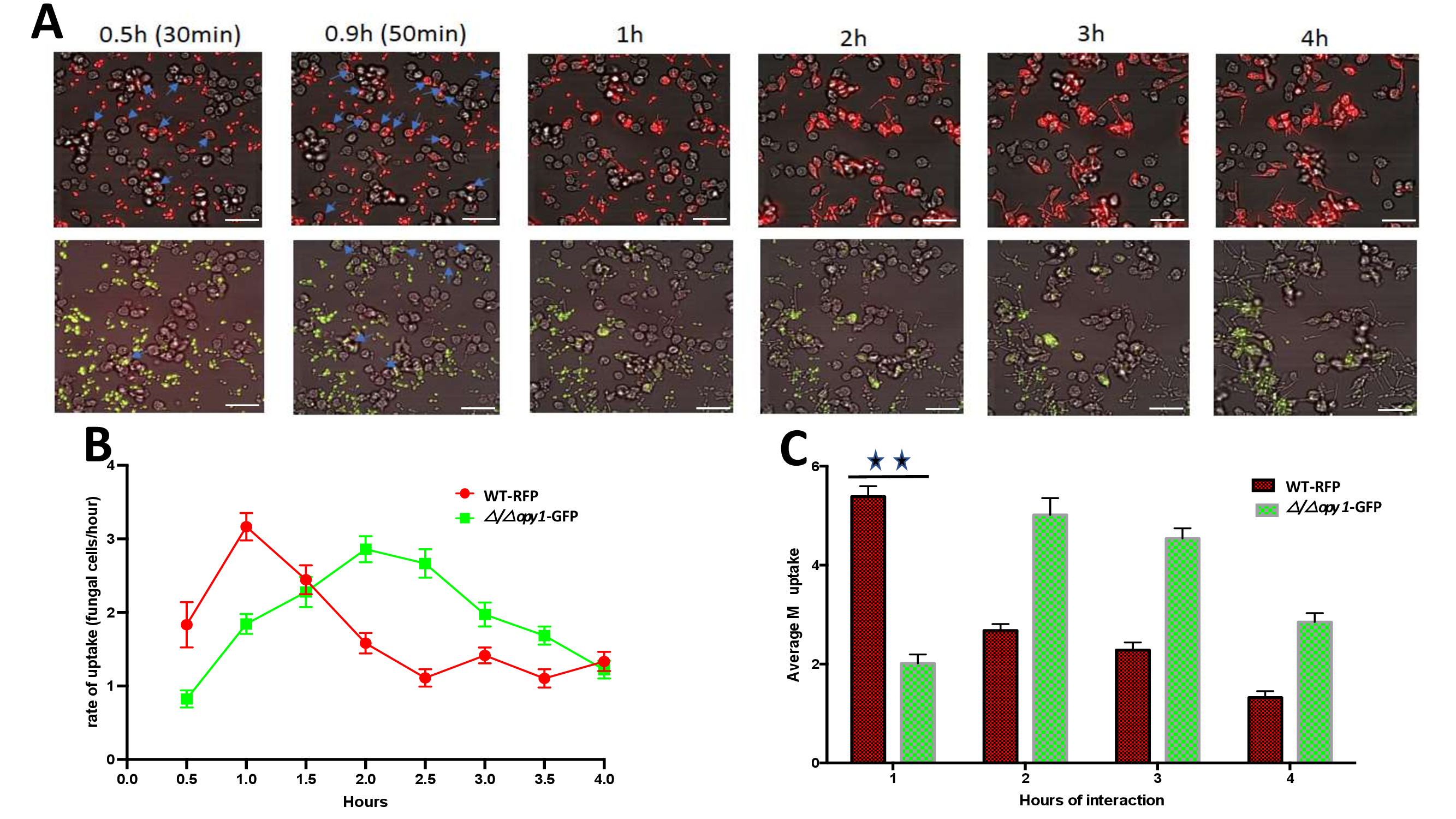

Supplement: Supplementary Figure 1 — The kinetics of engulfment displayed by macrophages of ΔΔ/opy1-GFP (green) and WT-RFP (red) separately. [file Image_1.TIFF]

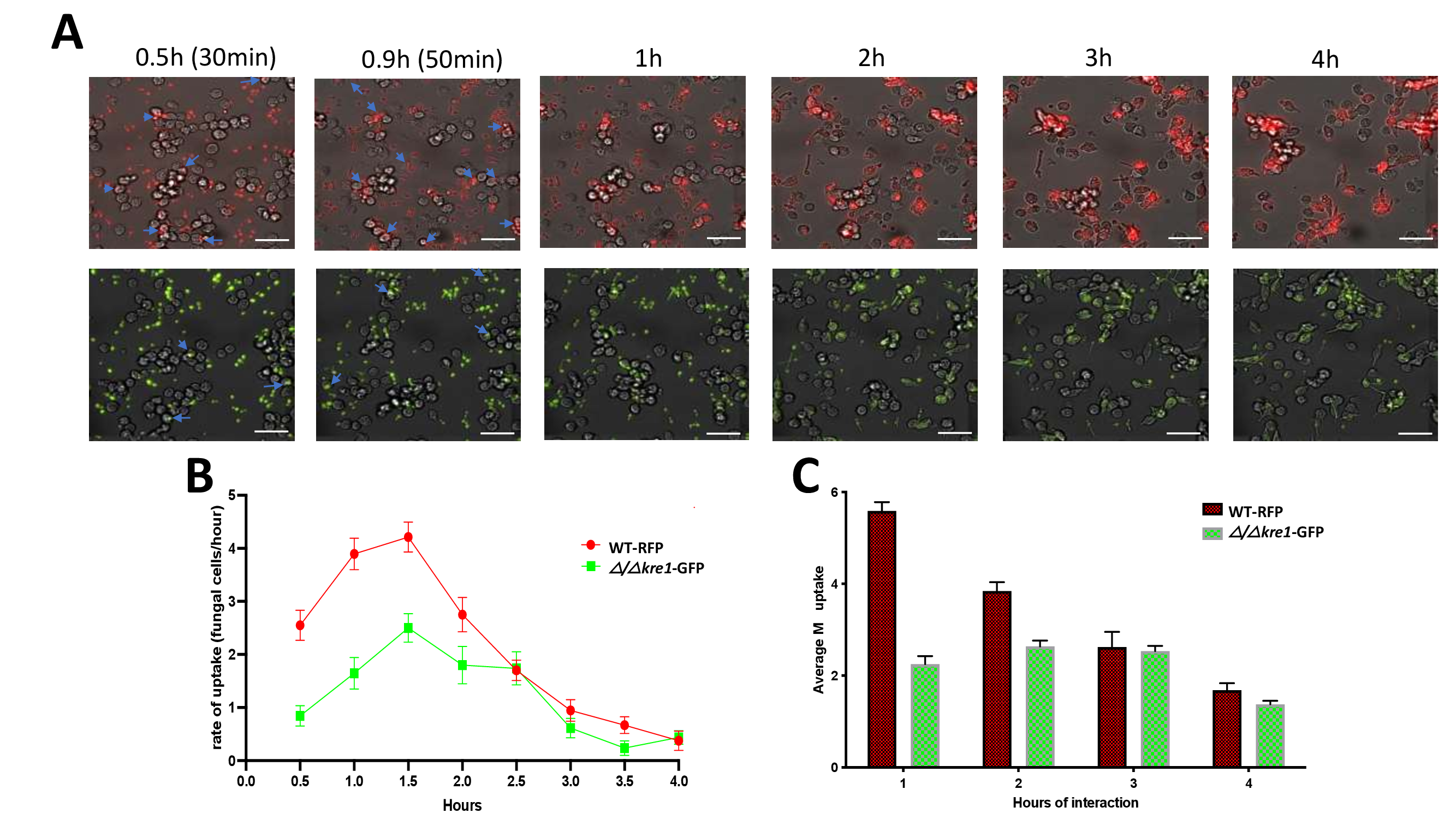

Supplement: Supplementary Figure 2 — The kinetics of engulfment displayed by macrophages of ΔΔ/kre1-GFP (green) and WT-RFP (red) separately. [file Image_2.TIFF]
